# Genetic modulation of protein expression in rat brain

**DOI:** 10.1016/j.isci.2025.112079

**Published:** 2025-02-21

**Authors:** Ling Li, Zhiping Wu, Andrea Guarracino, Flavia Villani, Dehui Kong, Ariana Mancieri, Aijun Zhang, Laura Saba, Hao Chen, Hana Brozka, Karel Vales, Anna N. Senko, Gerd Kempermann, Ales Stuchlik, Michal Pravenec, Joseph Lechner, Pjotr Prins, Ramkumar Mathur, Lu Lu, Kai Yang, Junmin Peng, Robert W. Williams, Xusheng Wang

**Affiliations:** 1 Department of Neurology, University of Tennessee Health Science Center, Memphis, TN 38163, USA; 2 Department of Genetics, Genomics and Informatics, University of Tennessee Health Science Center, Memphis, TN 38163, USA; 3 Department of Structural Biology, St. Jude Children’s Research Hospital, Memphis, TN 38105, USA; 4 Department of Pharmaceutical Sciences, University of Colorado Denver, Aurora, CO 80045, USA; 5 Department of Pharmacology, Addiction Science, and Toxicology, University of Tennessee Health Science Center, Memphis, TN 38103, USA; 6 Institute of Physiology of the Czech Academy of Sciences, Prague 14200, Czech Republic; 7 Genomics of Regeneration of the Central Nervous System, Center for Regenerative Therapies Dresden, Dresden University of Technology, 01307 Dresden, Germany; 8 Department of Developmental Neurobiology, St. Jude Children’s Research Hospital, Memphis, TN 38105, USA; 9 Human Technopole, Viale Rita Levi-Montalcini, 20157 Milan, Italy; 10 Department of Pediatrics and the Herman B Wells Center for Pediatric Research, Indiana University School of Medicine, Indianapolis, IN 46202, USA; 11 Department of Microbiology and Immunology, Indiana University School of Medicine, Indianapolis, IN 46202, USA; 12 Department of Geriatrics, School of Medicine and Health Sciences, University of North Dakota, Grand Forks, ND 58202, USA

**Keywords:** Biochemistry, Genetics, Neuroscience

## Abstract

Genetic variations in protein expression are implicated in a broad spectrum of common diseases and complex traits but remain less explored compared to mRNA and classical phenotypes. This study systematically analyzed brain proteomes in a rat family using tandem mass tag (TMT)-based quantitative mass spectrometry. We quantified 8,119 proteins across two parental strains (SHR/Olalpcv and BN-Lx/Cub) and 29 HXB/BXH recombinant inbred (RI) strains, identifying 597 proteins with differential expression and 464 proteins linked to *cis*-acting quantitative trait loci (pQTLs). Proteogenomics identified 95 variant peptides, and sex-specific analyses revealed both shared and distinct *cis*-pQTLs. We improved the ability to pinpoint candidate genes underlying pQTLs by utilizing the rat pangenome and explored the connections between pQTLs in rats and human disorders. Collectively, this study highlights the value of large proteo-genetic datasets in elucidating protein modulation in the brain and its links to complex central nervous system (CNS) traits.

## Highlights

• Profiling rat brain proteome reveals genetic regulation of protein expression
• Integrating proteome and phenotypic data links protein expression to complex traits in rats
• Identifying sex-specific pQTLs uncovers distinct protein regulations between sexes
• Linking rat pQTLs to human disorders highlights the translational potential

## Graphical abstract

**Figure:**
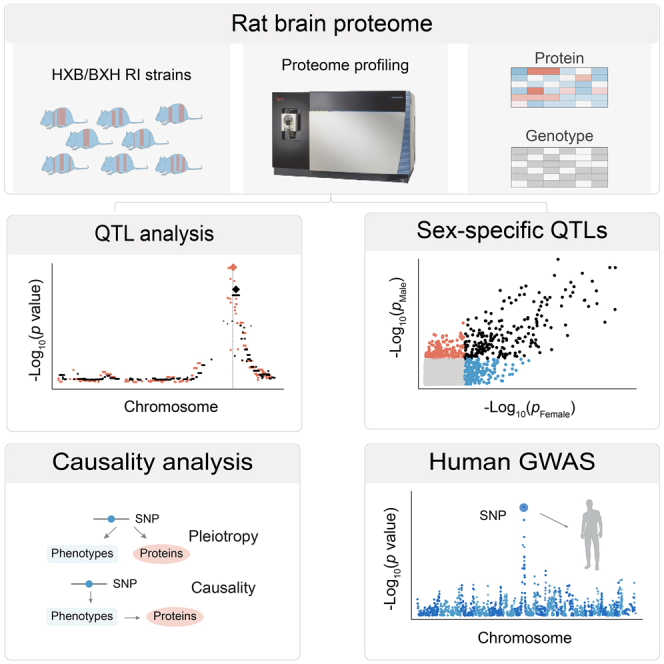

## Introduction

Genetic linkage analyses in rodents have unveiled numerous loci contributing to a diverse array of traits, including susceptibility to addictive behaviors, neurodegeneration, and hypertension.^1^^,^^2^^,^^3^ Despite these discoveries, linkage analyses alone fail to dissect the underlying biological processes causing these traits.^3^^,^^4^ However, a growing body of converging evidence strongly indicates that the associations between genomic loci and traits are primarily mediated through the genetic modulation of expression on molecular endophenotypes.^5^^,^^6^ The endophenotypes encompass a wide spectrum of molecules—mRNAs, proteins, and metabolites—that serve as intermediates in complex cascades that link DNA variants to higher order traits. The accuracy and throughput with which molecular endophenotypes can now be quantified in larger populations and families hold significant promise in bridging the gap between genomic loci and traits.^7^^,^^8^

Almost all work on the genetics of expression and its linkage to higher order traits has been involved in mRNA expression first using arrays and now using RNA sequencing (RNA-seq).^9^^,^^10^^,^^11^ In contrast, much less is known about the arguably more functionally relevant variation in the genetic control of protein expression and its causal contributions to higher order traits. Variations in mRNA expression often do not reliably reflect variations in protein expression due to intricate post-transcriptional and translational regulatory mechanisms.^12^^,^^13^^,^^14^^,^^15^ As the main driver of cellular function,^16^ variation in protein levels should have a more direct impact on cell and tissue function. Consequently, unraveling the genetic variation and the genetic control of protein levels holds great potential to dissect molecular and cellular mechanisms underlying the variation in traits.

To investigate the genetic basis of trait variation in molecular endophenotypes, genetic reference populations such as the mouse BXDs,^17^^,^^18^^,^^19^ Collaborative Cross,^20^^,^^21^ and the HXB/BXH recombinant inbred (RI) family^22^^,^^23^ have been used extensively over the past decade. However, there has been a surprising lack of brain proteomic work in these animal models.^24^ The HXB/BXH family is the largest and most deeply phenotyped set of genetically diverse but fully inbred set of rats. It has been used mainly to dissect loci genetic variants that modulate metabolic and cardiovascular diseases,^23^^,^^25^^,^^26^^,^^27^ but in recent work this family is increasingly being used to study the genetics of substance use disorders.^28^^,^^29^^,^^30^ All members of the family have been fully sequenced, and high-density marker maps of all classes of DNA variants are now available for both forward and reverse genetic analyses.^31^^,^^32^ There are also extensive transcriptomic datasets for several systems,^33^^,^^34^^,^^35^ but now for the first time also for the brain.^28^ Finally, there is extensive and curated phenotypic data for this family in GeneNetwork.org,^36^^,^^37^^,^^38^ and this enables on-line mapping, as well as integrative analyses to explore potential networks linking loci to traits.

In this study, we systematically quantify the variation and genetic architecture of protein expression in whole brain and behavioral traits in active place avoidance as a task critically dependent on the hippocampus in the rat HXB/BXH RI strains. We start by generating proteome across 29 HXB/BXH RI strains, in conjunction with the two parental strains, SHR and BN-Lx. We then quantify sequence and expression variations at the protein level between the two parental strains using a proteogenomics approach. We identify both *cis* and *trans-*acting loci contributing to variation in protein expression in the brain. We zoom in on gene candidates using pangenome tools. Finally, we explore relations between gene loci, mRNA and protein expression, and phenotypic traits.

## Results

### Proteomic profiling and analysis of the HXB/BXH RI rat family

**Figure 1:**
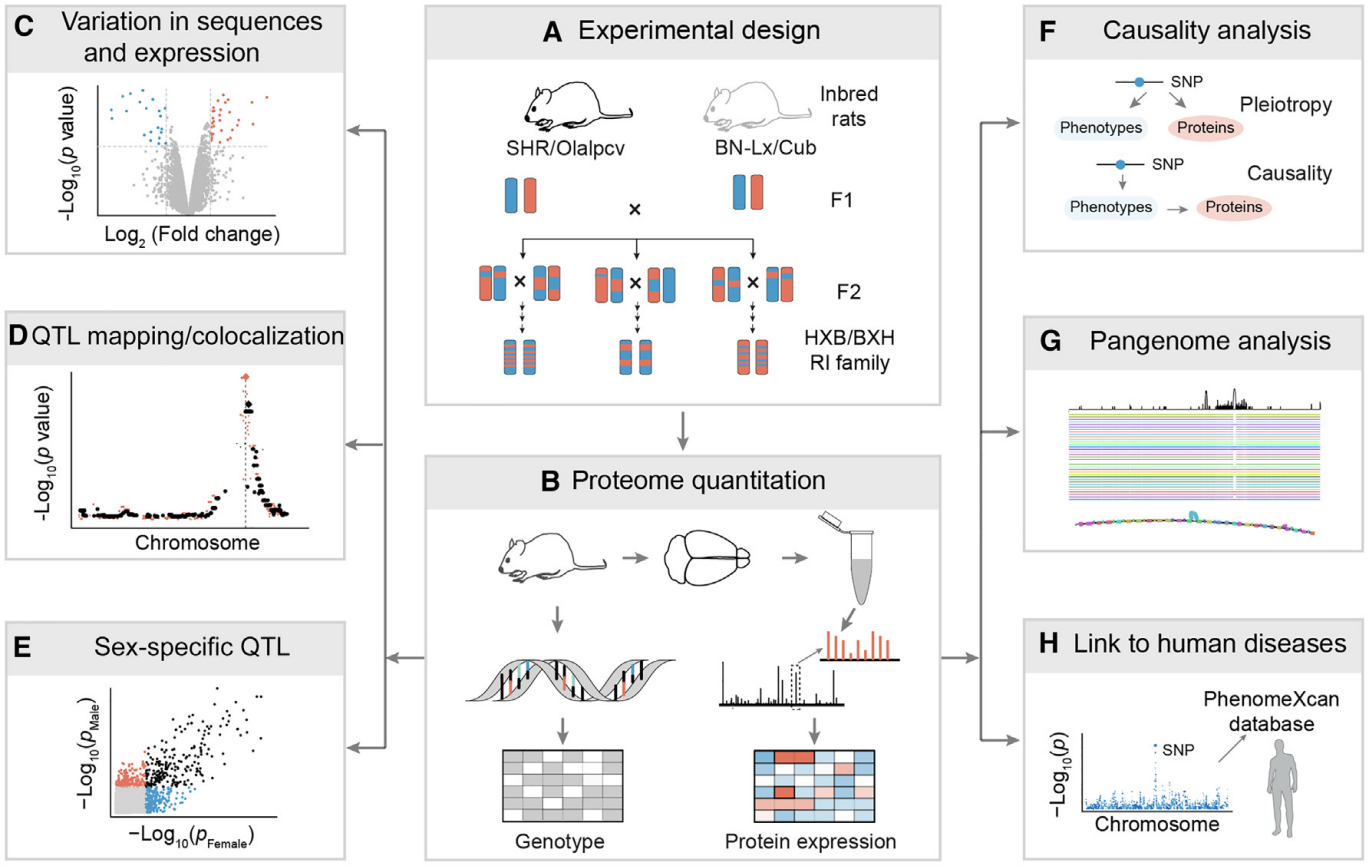
Experimental design and analysis pipeline employed in this study (A) Schematic representation illustrating the inclusion of postmortem brain samples from 62 participants, encompassing 29 HXB/BXH R1 strains alongside their respective parental strains. (B) Profiling of the deep brain proteome was conducted using 11-plex/16-plex TMT-based proteomics, followed by rigorous quality control and comprehensive data analysis. The brain proteomic data, along with corresponding genotype data, were meticulously prepared for subsequent linkage analysis. (C) Volcano plot demonstrates the differential expression of proteins. (D) QTL analysis was employed to identify genetic regulation of protein expression or gene expression, colocalization *cis*-pQTL and *cis*-eQTL, or *cis*-pQTL and *trans*-pQTL. (E) QTL analysis employed to identify genetic regulations of protein expression in different sex. (F) Integrative analysis to link pGenes to phenotypes. (G) Integrative analysis to link pGenes to phenotypes. (H) Pangenome analysis to explore the variation across all mapping strains.

**Figure 2:**
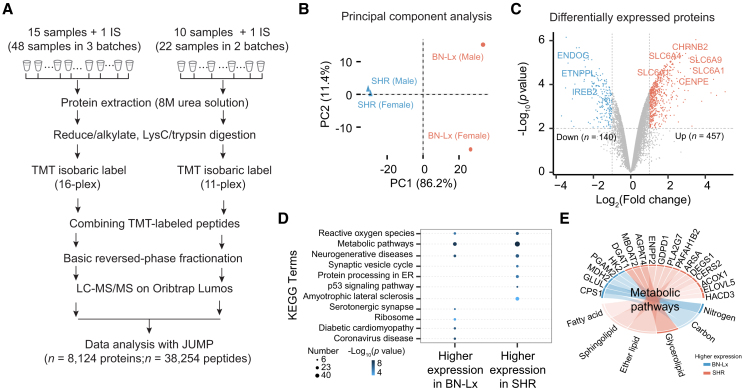
Deep profiling of rat brain proteome (A) Workflow of 11-plex/16-plex TMT-based proteome analysis. LC-MS/MS was performed on a total of 10/15 samples, along with 1 internal standard (i.e., 10/15 pooled samples). MS raw data were analyzed using JUMP software. (B) Principal-component analysis (PCA) of all quantified proteins. (C) Volcano plot displaying the differentially expressed proteins. Log_2_ fold change was plotted against the −log_10_ adjusted *p* value with one, criterion: 2-fold change and 1% adjusted *p* values. (D) KEGG pathways enrichment analysis of DEPs highly expressed in SHR or BN-Lx strain. Each bubble represents a KEGG term, positioned based on its enrichment score (x axis) and significance (y axis). Bubble size corresponds to the number of genes associated with the KEGG term, and color intensity indicates the significance level (*p* value). Highly enriched processes are represented by larger, more intensely colored bubbles. (E) Strain-specific sub-pathway enrichment in metabolic pathways. The circle plot illustrates the associated proteins within the metabolic sub pathways. Proteins with higher expression in the BN-Lx strain are highlighted in blue, while those with higher expression in the SHR strain are highlighted in red.

In our study, we explored genetic variation in protein expression, identified protein expression quantitative trait loci (pQTLs), uncovered sex-specific pQTLs, assessed the impact of pQTLs on phenotypic traits, revealed causal variants within the rat pangenome, and investigated potential links between rat pQTLs and human disorders (Figure 1). For the proteomics experiments, we employed two batches of 11-plex and three batches of 16-plex tandem mass tags (TMT) coupled with two-dimensional liquid chromatography-tandem mass spectrometry (LC-MS/MS) strategy (Figure 2A). In this analysis, we identified and quantified a total of 11,118 proteins in at least one batch at the protein false discovery rate (FDR) < 1%. Out of these, 8,124 (73.07%) proteins were detected across all biological 62 samples (Figures S1 and S2A; Table S1). We performed extensive quality control, including sample identity verification using SMAP, a program for sample verification and correction^16^ and batch effect removal using the limma R package.^39^ Principal-component analysis (PCA) showed that two replicates of the two parental rat strains grouped well (Figure 2B). Pearson correlation analysis showed that the two replicates of each strain displayed a high correlation (*r* > 0.99; Figure S2B). Paired t test of 31 male-female littermates revealed that only 60 (0.7%) showed differences between sexes (*p* < 0.01; before adjusting for multiple testing correction) (Figure S2C). In contrast, the two parental strains exhibited a large variation in protein expression (Figure S2D).

As the transcriptomic data had previously been generated for the HXB/BXH RI strains,^40^ we compared our proteomic data to transcriptomic findings. The quantified proteins represent approximately 50% of the transcripts identified in the transcriptomic data (Figure S2E). A moderate positive correlation between expression levels of mRNAs and proteins (*r* = 0.35) was observed (Figure S2F), which is consistent with previous findings in human study.^24^

### Genetic variation in sequences and expression levels

Our deep proteome provides an opportunity to investigate sequence variations between the two parental strains at the protein level. To identify variant peptides with the SHR allele, we performed proteogenomic analysis using JUMPg.^41^ We identified a total of 95 variant peptides at a peptide FDR of 1% (Table S2A). Of these variant peptides, we also detected 61 corresponding reference peptides with the BN-Lx reference allele.

To further examine whether the expression levels of these variant peptides change, we identified a total of 78 variant peptides with statistically significant changes (FDR < 1%; Table S2A). Of these variant peptides, we also detected 51 corresponding reference peptides with the BN-Lx reference allele. As expected, all variant peptides showed significantly higher expression in SHR compared to BN-Lx, as illustrated with two variant peptides (Figure S2G; Table S2A). Eight missense variants were predicted deleterious by the Sorting Intolerant from Tolerant (SIFT) program in ensembl web^42^ (Table S2A).

To measure variations in protein expression between the two parental strains of the HXB/BXH RI family—SHR and BN-Lx strains—we conducted a differential protein expression analysis using the limma R package. We identified a total of 597 differentially expressed proteins (DEPs) with an adjusted *p* value of 0.01 and a log_2_ fold change (log_2_FC) of 1 or greater (Figure 2C; Table S2B). Among these, 457 proteins displayed higher expression in the SHR strain, while 140 proteins exhibited higher expression in the reference BN-Lx strain. Kyoto Encyclopedia of Genes and Genomes (KEGG) enrichment analysis revealed that DEPs with higher expression in the SHR strain were significantly enriched in pathways related to reactive oxygen species (ROS), the synaptic vesicle cycle, and amyotrophic lateral sclerosis (ALS) (Figure 2D; Tables S2C and S2D). Notably, some pathways, such as those related to metabolism, were enriched in proteins highly expressed in both SHR and BN-Lx strains. To determine if subpathways within the same pathway are differentially involved, we found that the BN-Lx strain shows enrichment in proteins associated with amino acid metabolism, including those involved in carbon and nitrogen metabolism. In contrast, the SHR strain exhibits higher expression of proteins involved in lipid and fatty acid metabolism, including the fatty acid, sphingolipid, ether lipid, and glycerolipid pathways (Figure 2E).

A subset of proteins—391 out of 8,268—exhibited remarkably high variability, defined as two standard deviations above the average coefficient of variation (CV, mean = 1.2 × 10^−2^; SD = 8.6 × 10^−3^) (Figure S2H; Table S1). These highly variable proteins were significantly enriched in pathways associated with serotonergic synapse (including CYP2D10, GNG4, CASP3, CYP2D3, GNG8, CYP2D1, PLCB2, and CYP2D26), oxidative phosphorylation (including NDUFA8, NDUFAB1, COX17, ATP5PO, CYCT, ATP6V0C, ATP5F1E, and COX6B1), and inflammatory mediator regulation of TRP channels (such as PRKCH, MAP1, ASIC2, PLCB2, and KNG1) (Figure S2I; Table S2E).^43^^,^^44^^,^^45^^,^^46^

**Figure 3:**
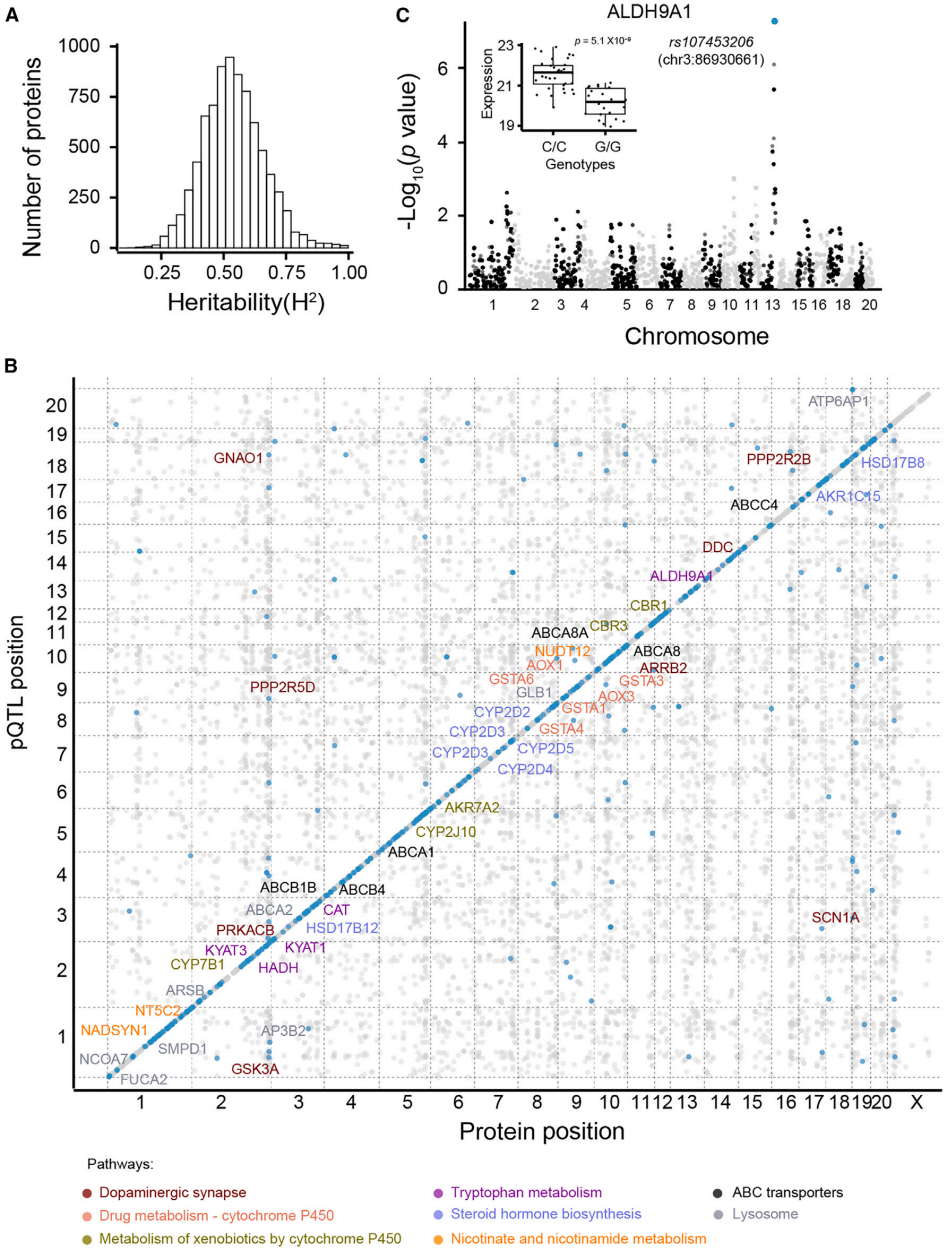
Genetic modulation of the brain proteome (A) Genetic variance and heritability estimate for all 8,119 identified proteins. (B) Genome-wide mapping plot displaying the physical protein location in total megabases versus QTL genetic location. (C) Manhattan plot showing a *cis*-pQTL (i.e., *rs107453206*) is associated with ALDH9A1 protein expression.

We next sought to estimate the heritability (*h*^*2*^) of protein expression, which is defined as the proportion of additive genetic variation contributing to the overall observed variation. The analysis revealed that the protein heritability in the HXB/BXH RI family has a median heritability of 0.54, ranging from 0.08 to 0.99 across all expressed proteins (Figure 3A; Table S1). For example, 2,4-dienoyl-CoA reductase 1 (DECR1) showed a high heritability of 0.94. The high heritability of proteins in the HXB/BXH family makes them particularly amenable to pQTL mapping.

### Genetic modulation of protein expression

We proceeded to investigate the genetic modulation of protein expression by conducting a comprehensive proteome-wide linkage analysis. Leveraging data from 8,119 proteins detected in 29 HXB/BXH RI strains and 10,465 genotypes, we identified 318 *cis*-acting (or local acting) loci that modulate the expression of 464 cognate proteins at a cutoff value *p* < 1.01 × 10^−3^ (see STAR Methods, Figure 3B, and Table S3A) using the GEMMA mapping software.^47^ These *cis*-pQTLs were enriched for metabolic pathways, such as drug and amino acid metabolisms. For example, *cis*-pGenes such as AOX3, CAT, KYAT3, AOX1, KYAT1, HADH, and ALDH9A1 were enriched in the tryptophan metabolism (Figure 3B). As an example, we found that ALDH9A1 (UniProt ID: Q9JLJ3) had a *cis*-pQTL with a linkage score *p* value of 4.76 × 10^−8^ (Figure 3C), located on chromosome 13 at 86,930,661 bp (*rs107453206*). Comparing the alternative allele (C/C) with the reference allele (G/G), we observed a significant increase in protein expression (20.13 vs*.* 21.56, *p* = 5.10 × 10^−9^) (Figure 3C inset). ALDH9A1 is highly expressed in the brain relative to other tissues^48^^,^^49^ and plays a role in an alternate biosynthesis pathway of gamma-aminobutyric acid (GABA) by catalyzing the oxidation of *γ*-aminobutyraldehyde.^49^^,^^50^^,^^51^

**Figure 4:**
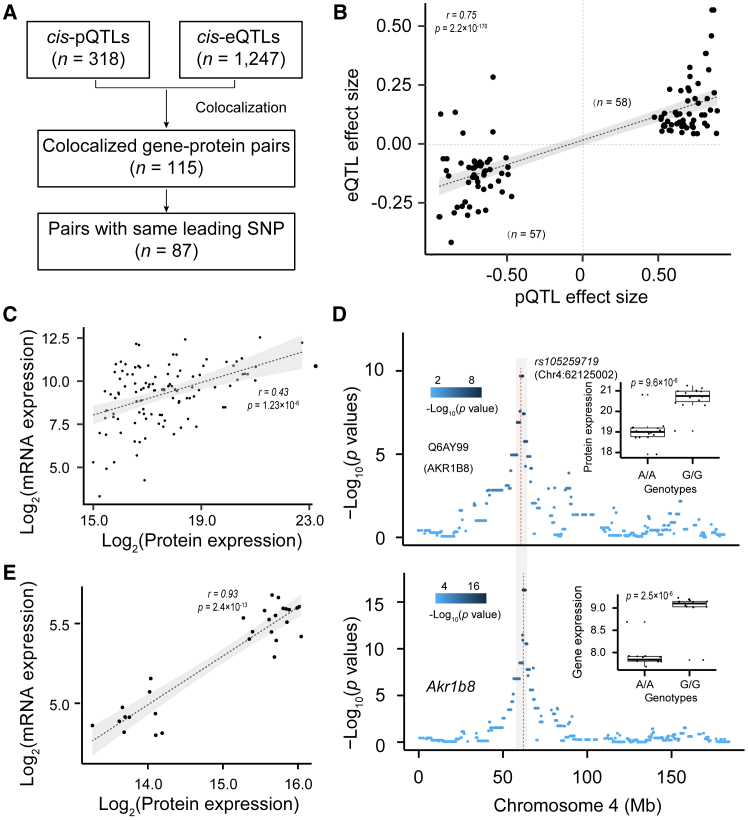
Colocalization of QTLs regulating the expression level of genes and proteins (A) Workflow showing colocalization analysis and number of genes colocalized by *cis*-eQTLs and *cis*-pQTLs. (B) Scatterplot showing the distribution of effect sizes of colocalized *cis*-eQTLs and *cis*-pQTLs. (C) Correlation of expression levels of colocalized *cis*-eGenes and *cis*-pGenes. (D) Manhattan plot showing a colocalized QTL modulating *Akr1b8* gene and protein expression. (E) Correlation of expression levels of colocalized *cis*-eGenes and *cis*-pGenes.

To explore the linkage between mRNA and corresponding protein expression levels, we utilized 18,278 gene expression quantitative trait loci (eQTLs) from the brain RNA-seq data of 30 HXB/BXH RI cases.^40^ We identified 1,247 *cis*-eQTLs (Table S4) with acceptance cutoff thresholds (*p* = 3.00 × 10^−3^; see STAR Methods). We compared these with 318 *cis*-pQTLs and found 115 pairs of colocalized QTLs (Figure 4A; Table S5A). The vast majority (75.6%; 87/115) of these colocalized mRNA-protein loci showed the same allelic effect polarity, with a mean Pearson correlation coefficient (*r*) of 0.78 (*p* value = 2.2 × 10^−170^; Figure 4B). This strongly supports the notion that an increase in the effect size of *cis*-eQTLs is indicative of an increase in that of *cis*-pQTLs. In contrast, we observed only a moderate correlation of expression levels between colocalized proteins and genes (*r* = 0.43; *p* value = 1.23 × 10^−6^; Figure 4C). For example, AKR1B8 (UniProt ID: Q6AY99) was identified as a significant *cis*-pQTL (*p* value = 2.03 × 10^−10^), and a significant *cis*-eQTL (*p* value = 5.50 × 10^−18^) (Figure 4D), supported by a significant positive correlation (*r* = 0.93) of expression at the gene and protein levels (Figure 4E).

### Sex-specific modulation of protein expression

To investigate the impact of sex on protein expression and its genetic regulation, we employed male and female protein expression data to conduct a proteome-wide linkage analysis. Utilizing expression levels of 8,119 proteins from HXB/BXH RI strains with both male and female, we identified 305 *cis*-acting loci in females modulating the expression of 433 proteins (Table S3B, with a cutoff value of *p* < 3.33 × 10^−3^), and 296 *cis*-acting loci in male influencing the expression of 405 proteins (Table S3C), with a cutoff value of *p* < 2.85 × 10^−3^ (see STAR Methods). Of these, 219 proteins with *cis*-pQTLs overlapped between males and females, while 214 proteins were identified exclusively in females and 186 proteins exclusively in males (Figure S3A). Furthermore, when comparing these findings with 318 proteins identified from sex-averaged expression levels, we observed 134 (42%) proteins that were shared by females, males, and sex-average (Figure S3B). Enrichment analysis revealed enrichment in multiple sex-related pathways, such as the regulation of peptide hormone secretion and intracellular estrogen receptor signaling pathways. For instance, in male, the expression of PFKL (phosphofructokinase liver type) in the modulation of peptide hormone secretion pathway is controlled by a locus on chromosome 20 (*rs64542764*, Chr20:10,518,616), with a mapping *p* value of 3.46 × 10^−4^ (Figure S3C). PFKL has previously been associated with sex differences in the metabolism of glucose and glycerol.^52^ In females, the expression of striatin 3 (STRN3) is controlled by a locus on chromosome 6 (*rs105312859*, Chr6: 73,779,782) with a mapping *p* value of 9.46 × 10^−5^ (Figure S3D). This observation is supported by an earlier finding that a splicing variant of STRN3 associates with estrogen receptor-alpha (ERα) in a ligand-dependent manner.^53^ Additionally, STRN3 has also been shown to be significantly upregulated in female breast cancer.^54^ These findings underscore the critical role of sex-specific genetic factors in shaping protein expression patterns.

### *Trans*-pQTLs modulate expression of downstream proteins

**Figure 5:**
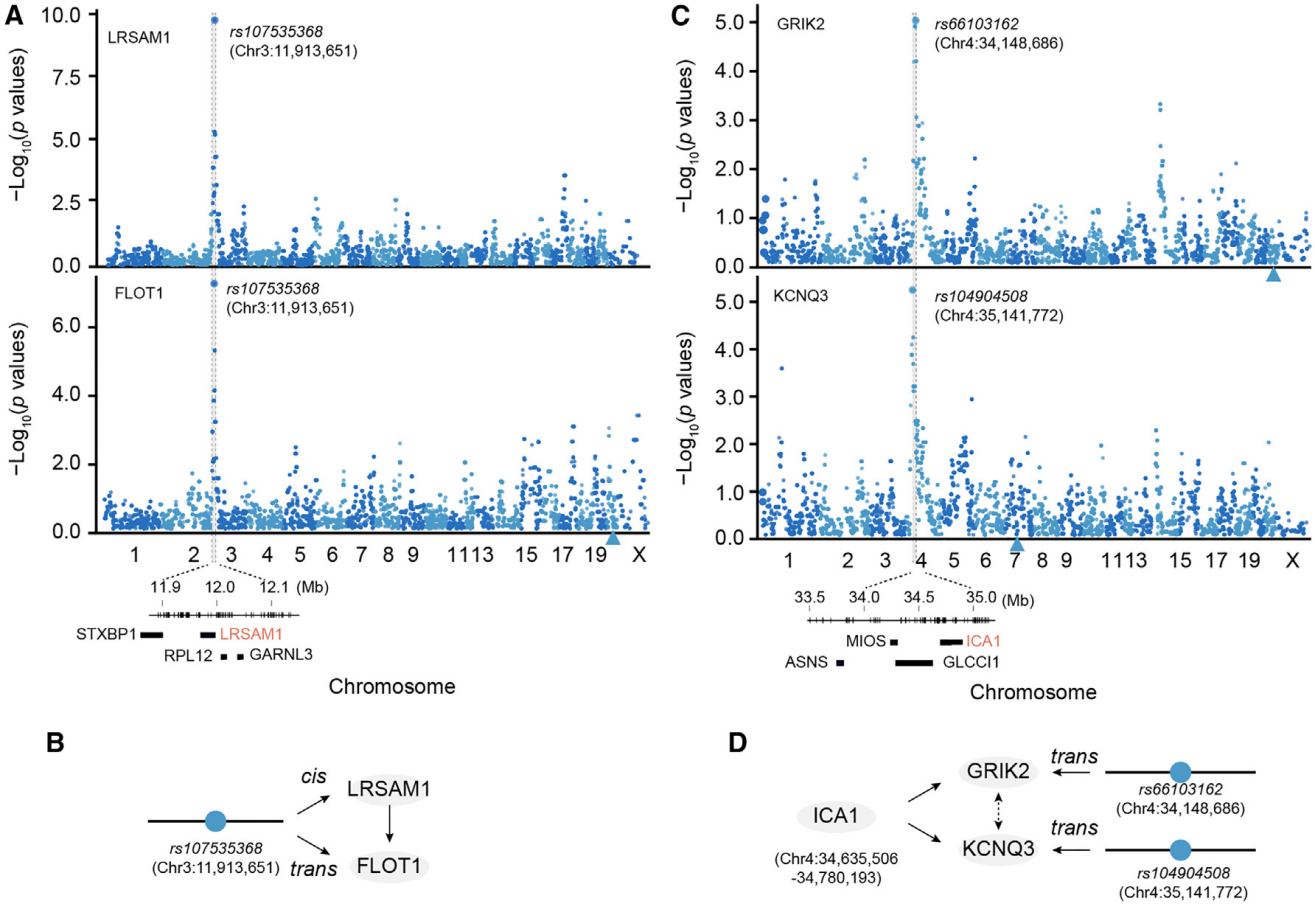
Example of genetic regulation of a protein with *trans*-pQTL (A) Manhattan plot showing an SNP (*rs107535368*) reveals its association with LRSAM1 protein expression through *cis*-regulation, and simultaneously *trans*-regulates FLOT1 protein expression. The x axis represents genomic positions, while the y axis denotes the −log_10_-transformed *p* values of the association. The QTL region is highlighted in the gray box, covering four candidate genes, including LRSAM1 protein. (B) Schematic diagram showing the regulation of LRSAM1 abundance by a *cis*-pQTL, which regulates FLOT1 protein expression level as a *trans*-QTL. (C) Manhattan plot showing a *trans*-pQTL (i.e., *rs66103162*) associated with GRIK2 protein expression and another *trans*-pQTL (i.e., *rs104904508*) associated with KCNQ3 protein expression. (D) Schematic diagram illustrating the causal relationships between the SNP, ICA1, GRIK2, and KCNQ3.

To determine whether a *trans*-pQTL regulates the expression of distant proteins through its colocalized *cis*-pQTL, we conducted a linkage analysis for *trans*-pQTLs (>10 Mb away from the regulated protein on the same chromosome or different chromosomes), identifying 87 *trans-*acting loci that modulate the expression level of 116 proteins (Table S3A, *p* value <10^−5^). By colocalizing these 87 *trans*-pQTLs with 318 *cis*-pQTLs, we found 45 pairs of colocalized QTL signals (Table S5B). One colocalized QTL is located on chromosome 3 near the marker *rs107535368* (Chr3:11,913,651 bp). This locus regulates the expression of FLOT1 (flotillin 1) as a *trans*-pQTL, with a mapping *p* value of 1.43 × 10^−7^, and the expression of LRSAM1 (i.e., leucine rich repeat and sterile alpha motif containing 1) as a *cis*-pQTL, with a mapping *p* value of 1.48 × 10^−10^ (Figure 5A). Despite the absence of an apparent functional link between LRSAM1 and FLOT1, LRSAM1 emerges as a plausible candidate protein regulating the protein expression of FLOT1 (Figure 5B).

Another notable *trans*-pQTL example is a locus on chromosome 4 regulating the expression of GRIK2, with a mapping *p* value of 8.09 × 10^−6^, and another locus on chromosome 4 regulating the expression of KCNQ3, with a mapping *p* value of 5.61 × 10^−6^ (Figure 5C). By examining the candidate proteins within the locus, we found that ICA1 is a compelling candidate protein regulating the expression of both GRIK2 and KCNQ3 (Figure 5D).

### Linking variation in protein expression to phenotypic traits

**Figure 6:**
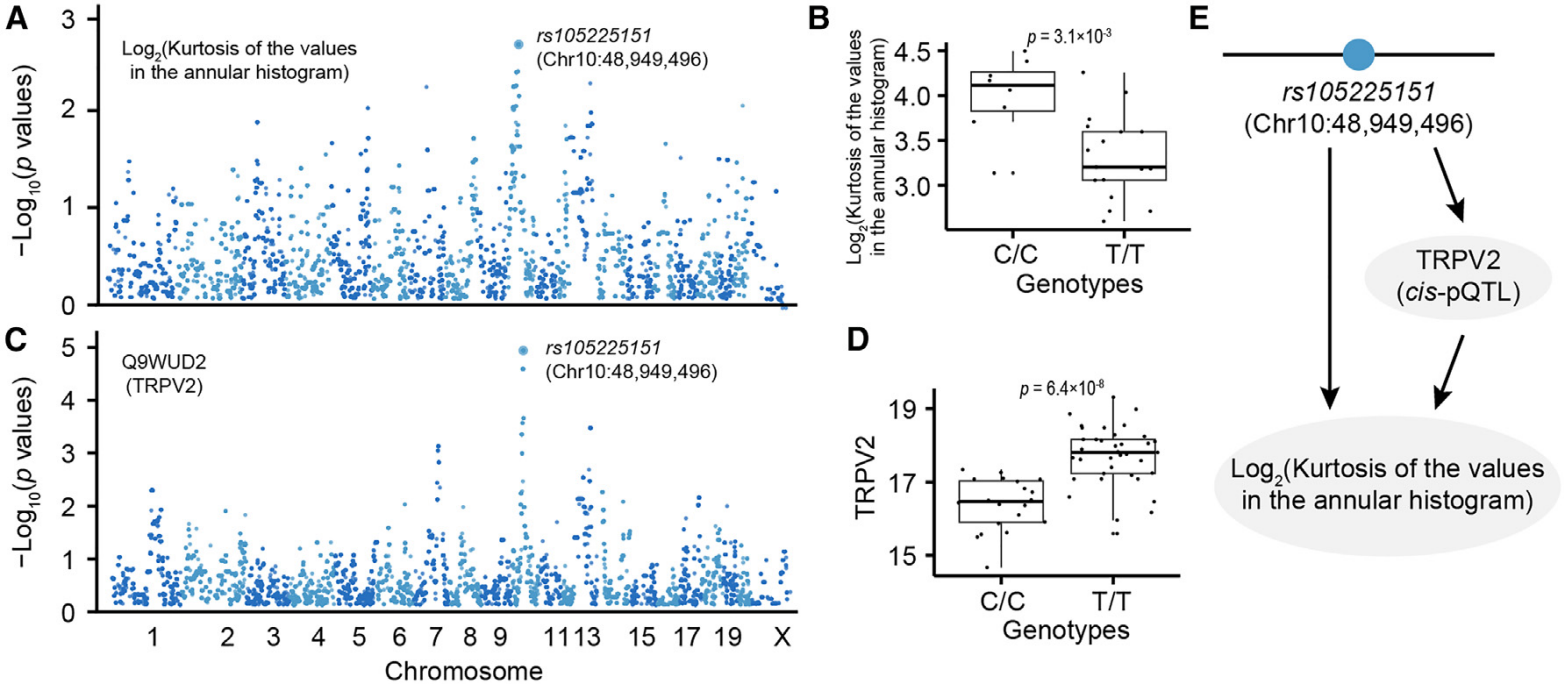
Association of proteins with *cis*-pQTL and behavioral traits (A) Manhattan plot showing the association of a QTL (i.e., *rs105225151*) with a behavioral trait: Kurtosis factor of annular-Gaussian beams. (B) Boxplot showing the trait values between reference allele and the alternative allele. (C) Manhattan plot showing a *cis*-pQTL (i.e., *rs105225151*) is associated with the TRPV2 protein. (D) Boxplot showing the expression levels between reference allele and the alternative allele. (E) Diagram illustrating a causal relationship between a genotype, TRPV2 expression level, and a behavioral trait.

To determine whether there is a potential causal association between pQTLs, as identified in this study, and phenotypic traits, we collected eight behavioral traits for the HXB/BXH family that are associated with spatial learning (Table S6). Spatial learning was assessed with an active place avoidance task with reversal (a dry-land spatial task), open-field, and beam-walking tests. Linkage analysis revealed that a locus (i.e., *rs105225151*; Chr10:48,949,496 bp) is significantly associated with Kurtosis factor of annular-Gaussian beam, with a mapping *p* value of 1.86 × 10^−3^ (Figures 6A and 6B). The locus was also found to be associated with the expression level of TRPV2 protein, with a mapping *p* value of 7.09 × 10^−6^ (Figure 6C). The reference allele has 1.72-fold (20.24 vs*.* 19.46 on log_2_ scale) higher expression than the alternative allele (Figure 6D). A Bayesian network analysis revealed that this locus potentially has a causal effect on both protein expression and phenotype (Figure 6E) using the Bayesian Network Webserver (BNW) program.^55^^,^^56^

We further explored the link between 464 proteins with *cis*-pQTL and 234 phenotypic traits in GeneNetwork database^36^^,^^37^^,^^38^ that were previously collected for the HXB/BXH family. Among these 234 phenotypic traits, 68 traits showed significantly associated with genomic loci (likelihood ratio statistic (LRS) score > 13.8; logarithm of odds (LOD) > 3). Using the coloc program, we found two proteins with significant *cis*-pQTLs that colocalized with phenotypic QTLs (Table S7). One of the two signals shared the same leading SNP. This SNP (i.e., SNP: *rs107228843*; Chr4: 11,422,002 bp) was found to be associated with a phenotype of basal glucose uptake (GN trait ID: 10082) (Figure S4A), with a mapping LRS of 21.95. Another SNP (i.e., SNP: *D4Rat7*; Chr4: 11,782,356 bp) was found to be associated with the expression level of N-acyl phosphatidylethanolamine phospholipase D (NAPEPLD), with a mapping *p* value of 1.19 × 10^−6^ (Figure S4B). The expression level of NAPEPLD exhibited a high correlation with the levels of basal glucose uptake (Figure S4C). A causality analysis revealed that this locus potentially has a pleiotropic effect on both protein expression and phenotype (Figure S4D) using the BNW program.^55^^,^^56^

### Pangenome analysis improves the identification of causal variants in QTLs

One commonly employed approach for identifying candidate genes within a QTL interval involves the identification of functional variants between two parental strains in a mapping population, such as the BXH/HXB panel. However, the approach is typically limited in resolution as it relies on mapping markers from the two parent strains. A better alternative is to leverage the pan-genome, which encompasses genome sequences for all HXB/BXH RI strains, to explore the variation across all mapping strains. To demonstrate candidate gene discovery using the pangenome, we focused on a small subset of proteins—six proteins with *cis*-pQTLs believed to influence phenotypic traits—for pangenome analysis. Among these, Fah (fumarylacetoacetate hydrolase) emerged as an intriguing example. Fah is mapped as a *cis*-pQTL with a mapping *p* value of 6.53 × 10^−5^ (Figure S5A). The QTL is colocalized with a phenotypic QTL modulating cysteine levels (Figure S5B). The expression level of Fah exhibited a high correlation with the levels of plasma cysteine (Figure S5C). To investigate functional variants within the Fah gene, we utilized PanGenome Graph Builder^57^ to obtain an unbiased graph representation^58^ of the pangenome of this gene and then applied the Optimized Dynamic Genome/Graph Implementation (ODGI)^59^ to understand its variation in the HXB/BXH family. Our analysis revealed a 142 bp insertion and deletion (INDEL) across the BXH/HXB strains (Figures S5D and S5E). The INDEL pattern is concordant with the expression of Fah across the strains. The rat pangenome enhances the discovery of candidate functional variants associated with the pQTLs and phenotypic variations.

To validate this finding, we employed FAH siRNA to reduce its expression in the SH-SY5Y neuronal cell line, and subsequently conducted proteomics experiments. As shown in Figure S5F, FAH siRNA led to a significant decrease of FAH expression in SH-SY5Y cells compared to control siRNA (*n* = 5 per group). Our analysis identified a total of 767 DEPs (adjusted *p* value < 0.05 and log_2_FC > 0.6) (Figure S5G). Among these, four proteins were associated with the cysteine and methionine metabolism pathway in KEGG, including three downregulated proteins: branched chain amino acid transaminase 1 (BCAT1), methylthioadenosine phosphorylase (MTAP) , and spermidine synthase (SRM). BCAT1 is an enzyme involved in cysteine metabolism through transamination reactions.^60^^,^^61^^,^^62^ This finding is consistent with a previous study in which FAH knockdown in the human melanoma cell line A375 resulted in the downregulation of 17 genes involved in the cysteine and methionine metabolism pathway (Figure S5H). Both studies demonstrated a reduction in BCAT1 expression in the FAH knockdown samples compared to controls (Figure S5I). Furthermore, a previous study showed that knock down of the BCAT1 protein resulted in a decrease in cysteine levels.^60^ Therefore, we believe that, although proteomics cannot directly measure cysteine levels, our experiment confirms that knocking down FAH in the SH-SY5Y cells reduces BCAT1 protein expression, which in turn affects cysteine levels.

### Genetic regulation of protein expression in rat points to human disorders

Rats, sharing extensive physiological and genetic similarities with humans, have served as a valuable model for translating protein expression variation insights from rodent studies to human disorders. We attempted to establish links between proteins with pQTL and human disorders. Using alcohol addiction as an example, we extracted 255 risk genes that are associated with “ever addicted to alcohol” from the PhenomeXcan database. We used the web of PhenomeXcan database in our study (http://apps.hakyimlab.org/phenomexcan/, release date: February 28, 2020). PhenomeXcan synthesizes 8.87 million variants from genome-wide association study (GWAS) on 4,091 traits with transcriptome regulation data from 49 tissues in GTEx v8 into an innovative, gene-based resource including 22,255 genes.^63^ Among these, one protein (GNAO1) was found to be *trans*-regulated by a locus (*rs64148360*) with a mapping *p* value of 7.97 × 10^−6^ (Figure S6A). GNAO1, a G protein subunit alpha o1, mediates the function of various neuronal receptors, including those implicated in alcohol addiction. This analysis highlights the translational potential of rat models in unraveling molecular mechanisms relevant to human disorders. Another intriguing example is the association between β-arrestin 2 (ARRB2) protein expression and alcohol addiction. The expression of ARRB2 is regulated by a locus on chromosome 10 (*rs105494285*, Chr10:58,370,362), with a mapping *p* value of 1.08 × 10^−5^ (Figure S6B).

## Discussion

In the present study, we aimed to elucidate the intricate mechanisms governing genetic modulation of protein expression in the rat brain and to understand its subsequent effects on phenotypic traits. To this end, we conducted a deep proteome profiling of the brain across 29 rat HXB/BXH RI strains, as well as their two parental strains. This analysis enabled us to delineate the genetic modulation landscape of protein expression in the rat brain. We investigated the association between pQTLs and behavioral traits and used pangenome analysis to identify potential genetic variants for pQTLs and phenotypic QTLs. Moreover, we explored the potential link between rat pQTLs and human disorders. Collectively, these findings contribute to our understanding of the genetic regulation of protein expression in the rat brain and its potential link to phenotypic variation.

We have provided what we believe to be the most comprehensive proteomic data for the HXB/BXH rat RI strains to date. This dataset offers a valuable resource to explore the variation in sequence, expression, and pathways between SHR and BN-Lx strains. Employing a proteogenomics approach, we have successfully identified 95 variant peptides carrying non-synonymous variants. This number is comparable to the findings reported in previous studies.^35^ In addition, we observed that the pathway associated with mitochondrial ROS is highly enriched in the DEPs between SHR and BN-Lx strains at the protein expression level, which is consistent with the previous finding that genomic variants between rat substrains, including SHR and BN-Lx, are also enriched in the ROS pathway.^64^

Genetic regulation of protein expression at the cell-type level enhances our understanding of the molecular mechanisms underlying complex traits and diseases. To address the limitations of our bulk-level analysis in this study, we also performed cell-type pQTL mapping by deconvoluting bulk proteomics data to better disentangle these complex regulations and provide more precise associations at the cell-type level. To estimate proteomic cell-type proportions, we used single-cell transcriptomics data from a previously published study,^65^ as single-cell proteome data are not available for rats. We estimated cell-type proportions using CIBERSORT,^66^ followed by sample-wise cell-type deconvolution using the bMind software.^67^ After obtaining sample-wise cell-type expression data, we conducted pQTL mapping for each cell type, identifying a total of 982 cell-type-specific QTLs, including 97 neuron-specific, 16 microglia-specific, 48 astrocyte-specific, and 14 oligodendrocyte-specific QTLs (Figure S7A). To explore the degree of colocalization between genetic variants linked to bulk protein expression and those specific to various cell types, we conducted a colocalization analysis for neuron and bulk proteomics using the coloc program. We observed a high consistency in the direction of effect sizes for pQTLs between neuron-specific and bulk proteomics (Figure S7B). For example, the seryl-tRNA synthetase 2 (SARS2) protein exhibited a strong posterior probability of colocalization (PP4 = 0.99). SARS2 expression was regulated by a *cis*-pQTL on chromosome 1, with a mapping *p* value of 8.87 × 10^−10^ in bulk data and 2.24 × 10^−8^ in neurons (Figure S7C).

This is one of the first studies that applies a rat pangenome in genetics. It is also one of the first studies to take pQTL and successfully use the pangenome to zoom in on underlying variants displaying genetic variants within the Fah gene, with an evident large insertion highlighted in SHR and some HXB strains that were never seen before. The emerging field of pangenomics allows the study of full genomes where the genome of all strains can be directly compared to each other. It provides the lossless comparison of all (complex) variants between individuals directly without the use of a single reference genome and inherited reference bias.^68^^,^^69^ This pangenomic approach will be especially powerful when individual genomes are all assembled from long-read sequence data, some of which are already available for the upcoming rat pangenome. This will enable a complete catalog of all genomic variants, including SVs and repeats, that differ between individuals and will be beneficial to future protein studies in rat.

In conclusion, our study offers a large-scale protein expression profile in the rat brain. We defined the genetic regulation of protein expression in the brain, emphasizing a wide range of exonic variants. We have successfully established causal links between identified protein pQTLs and phenotypic traits. Investigation of the possible connection between rat pQTLs and human disorders revealed insights that can inform translational studies, bridging the gap between animal models and human health. By elucidating the mechanisms of protein expression regulation, our work opens new pathways for discovering novel candidate genes associated with diverse phenotypes, contributing significantly to the field of genetics and neurobiology.

### Limitations of the study

The pQTL analysis in this study was conducted using a relatively modest number of strains (*n* = 29). This number is small, especially when compared to human studies and other rodent models, such as collaborative mouse crosses. Due to this small sample size, our study may face limitations in statistical power, potentially impacting the number of detected pQTLs. However, we envision significant improvements in our ability to capture a broader range of pQTLs with the inclusion of additional RI strains, such as the FXLE/LEXF panel. By expanding the diversity of strains analyzed, we aim to achieve a more comprehensive understanding of the genetic modulation of protein expression in the rat brain, overcoming the current limitations associated with the sample size. The expansion holds promise for unveiling a richer landscape of regulatory genetic elements influencing protein expression.

## Resource availability

### Lead contact

Further information and requests for resources should be directed to and will be fulfilled by the lead contact, Xusheng Wang (xwang39@uthsc.edu).

### Materials availability

This study did not generate new unique reagents.

### Data and code availability

• The mass spectrometry proteomics data have been deposited in the ProteomeXchange Consortium via the PRIDE^70^ repository (identifier: PXD058748). Additional data have been deposited on Zenodo (https://doi.org/10.5281/zenodo.14047911).
• The code developed for this study has been deposited on Zenodo (https://doi.org/10.5281/zenodo.14047911). All original code is publicly available as of the date of publication.
• Additional information required to reanalyze the data reported in this paper is available from the lead contact upon request.

## Acknowledgments

This work was supported by NIH/NIDA P30 Pilot Grant (1P30DA044223). L. Li and X.W. were also partially supported by NIH/NIA R01 (RF1AG072703) and NIH/NIDA R01 (R01DA056523). This study is also partially supported by the 10.13039/100000002National Institutes of Health grant RF1AG064909 awarded to J.P. M.P. is supported by the grant LUAUS25164 within the INTER-EXCELLENCE program of the Ministry of Education, Youth, and Sports of the Czech Republic and by a program from the National Institute for Research of Metabolic and Cardiovascular Diseases (Program EXCELES, ID Project No. LX22NPO5104) funded by the European Union–Next Generation EU; H.B., K.V., and A.S. were supported in part by the 10.13039/501100001824Czech Science Foundation (GACR) grants (21-16667K) and by the project 10.13039/100000056National Institute of Nursing Research (Program EXCELES, ID Project No. LX22NPO5107) funded by the European Union–Next Generation EU. We also acknowledge the University of Arkansas for Medical Sciences (UAMS) for providing the proteomic analysis under the IDeA National Resource for Quantitative Proteomics (R24GM137786).

## Author contributions

X.W., J.P., and R.W.W. planned and supervised all experiments and data analysis. L. Li, F.V., A.G., D.K., L. Lu, A.Z., G.K., A.S., and P.P. provided bioinformatics support and carried out data analysis. Z.W. and A.M. performed TMT-based proteomics experiment. L.S. and H.C. provided transcriptomic and genomic variant data, respectively. H.B., K.V., A.N.S., and M.P. conducted the behavioral testing and analyzed the results. J.L., R.M., and K.Y. conducted the knockdown experiment. L. Li, R.W.W., and X.W. wrote the manuscript.

## Declaration of interests

The authors declare no competing interests.

## STAR★Methods

### Key resources table

**Table:** 

REAGENT or RESOURCE	SOURCE	IDENTIFIER
**Experimental models**		

Rat	This study	https://doi.org/10.5281/zenodo.14047911

**Cell lines**		

SH-SY5Y cells	This study^71^^,^^72^	ATCC CRL-2266

**Deposited data**		

RNA-seq data	Saba et al.^40^	http://phenogen.ucdenver.edu
Mass spectrometry proteomics data	This study	PXD058748
Genotypic data	Jong et al.^43^	https://genenetwork.org/
Behavioral phenotype data	This study	https://doi.org/10.5281/zenodo.14047911
Behavioral phenotype data	GeneNetwork	https://genenetwork.org/
PhenomeXcan	Pividori et al.^63^	http://phenomexcan.org/
Single-cell transcriptomics data	Chen et al.^65^	NCBI GEO (accession number: GSE185538)
STRING database	Szklarczyk et al.^73^	https://string-db.org/

**Software and algorithms**		

R 4.04	R project	https://www.r-project.org/
Perl (v5.18.4)	Perl.org	https://www.perl.org/get.html
ANNOVAR	Yang et al.^74^	https://annovar.openbioinformatics.org/en/latest/user-guide/startup/
clusterProfiler R package	Yu et al.^75^	https://mrcieu.github.io/TwoSampleMR
LIMMA	Ritchie et al.^39^	https://www.bioconductor.org/packages/release/bioc/html/limma.html
JUMP software	X Wang et al.^76^	https://github.com/JUMPSuite/JUMP
JUMPg software	Li et al.^41^	https://github.com/gatechatl/JUMPg
GEMMA	Zhou et al.^47^	https://github.com/genetics-statistics/GEMMA
coloc	G Wang et al.^77^	https://github.com/cran/coloc
Pangenome Graph Builder	Garrison et al.^57^	https://github.com/pangenome/
CIBERSORT	Newman et al.^66^	https://cibersortx.stanford.edu/
bMind software	J Wang et al.^67^	https://github.com/randel/MIND
DIA-NN software	Demichev et al.^78^	https://github.com/vdemichev/diann
BNW	Ziebarth Et al^55^^,^^56^	compbio.uthsc.edu/BNW
UniProt	The UniProt Consortium^79^	https://www.uniprot.org/id-mapping
Code for this study	This study	https://doi.org/10.5281/zenodo.14047911

### Experimental model and study participant details

#### Animals

For this study, we utilized both male and female rats aged two months, from a total of 29 HXB/BXH RI strains and two parental strains. Regarding information related to animal strain, genotype, age, and sex provided in the link, https://doi.org/10.5281/zenodo.14047911. All experimental procedures involving animals were carried out in strict adherence to the guidelines approved by the Institutional Ethics Committees of the Institute of Physiology, Czech Academy of Sciences, located in Prague, Czech Republic (Permit number: 66/2014). No human participants were involved in this study. No additional data were collected from participants. No new participants were recruited for this study.

#### Cell lines

SH-SY5Y cells (ATCC), a human neuroblastoma cell line, were utilized in this study for knockdown experiments. The SH-SY5Y cells were cultured under standard conditions and transfected according to the manufacturer’s protocol to achieve optimal knockdown efficiency.

### Method details

#### Behavioral phenotyping

Male rats of the SHR/Ola and BN-Lx/Cub progenitor strains and 30 RI strains (being inbred for more than 90 generations) were obtained from the Department of Genetics of Model Diseases at the Institute of Physiology, Academy of Sciences of the Czech Republic. Number of rats in each strain ranged from 6 to 14. A total of 277 animals from 30 HXB/BXH strains were used, with 6–7 animals per strain included in the behavioral tests. The animals were housed in an air-conditioned animal room with a stable temperature (22°C), humidity (40%) and 12/12 light/dark cycle (light on at 7:00 a.m.). Animals were accommodated in the animal room for two weeks and behavioral testing was done at the age of 11–14 weeks. For testing in the active place avoidance task, requiring aversive reinforcement, conscious animals were gently implanted with a subcutaneous needle, which pierced the rat’s skin between its shoulders and its sharp end was cut and bent to form a small loop, preventing slip-out and providing purchase for an alligator clip delivering shocks. This procedure loads minimal stress onto animals and is analogous to subcutaneous injection in humans. Water and food were freely available to the rats throughout the study. All experimentation complied with current legislation on protection of laboratory animals (Animal Protection Code of the Czech Republic, European Union directives 2010/63/EC; 86/609/EEC and NIH guidelines).

The apparatus has been described previously in detail.^80^^,^^81^^,^^82^ Briefly, it consisted of a smooth metal disk (82 cm in diameter; elevated 1 m above the room floor), which rotated clockwise at 1 revolution per minute (rpm). A 50-cm-high transparent wall made of clear Plexiglas surrounded the arena. The animals had to avoid an unmarked, 60-deg to-be-avoided sector identified solely by its relationships to distal room cues. The position of the shock sector remained stable throughout each phase. The only change of the sector position occurred before the reversal phase.

The rats again wore a rubber jacket, which carried an infrared LED between animal’s shoulders. A computer-based tracking system (iTrack; Tracker, Biosignal Group, USA) located in an adjacent room recorded the rat’s position at a frequency of 25 Hz. Position series were stored for offline analysis (TrackAnalysis; Biosignal Group, USA). Whenever the rat entered the to-be-avoided sector for more than 300 ms, the tracking system delivered a mild electric shock (50 Hz, 0.5 s, 0.4–0.6 mA) and counted an entrance. If the rat did not leave the sector, additional shocks were given every 900 ms, but no more entrances were counted until the rat left the sector for more than 300 ms. Shocks were delivered through the implanted needle and the grounded arena floor (the highest voltage drop, and perception of shocks were between rats’ paws and floor). This shocking procedure was previously shown to be effective and safe for rats, leading to rapid avoidance behavior.^80^^,^^83^^,^^84^^,^^85^ The current was individualized for each rat to elicit a rapid escape response but to prevent freezing, however, in most cases, animals responded appropriately to 0.4 mA. There were no systematic differences between shock intensities between strains. After each rat, the floor was cleaned with ethanol, ensuring the rats could not use inter-trial scent marks. Rats were tested in semi-mixed design to avoid batch effects.

#### Protein extraction, quantification, and digestion

Whole rat brain tissues were weighed and homogenized using a lysis buffer composed of 50 mM HEPES (pH 8.5), 8 M urea, and 0.5% sodium deoxycholate, with a ratio of 100 μL of buffer per 10 mg of tissue. To inhibit phosphatase activity, a 1×PhosSTOP phosphatase inhibitor cocktail (Sigma-Aldrich) was added to the lysis buffer. The total protein concentration of each sample was determined using the BCA Protein Assay Kit (Thermo Fisher Scientific) and further confirmed through Coomassie staining of short SDS gels.^86^^,^^87^ Quantified protein samples (∼0.3 mg) in the lysis buffer containing 8 M urea were first subjected to proteolysis using Lys-C (Wako) at a ratio of 1:100 (enzyme to protein) at room temperature for a duration of 2 h. Subsequently, the samples were diluted 4-fold to reduce the urea concentration to 2 M, followed by digestion with trypsin (Promega) at a ratio of 1:50 (enzyme to protein) at room temperature overnight. To terminate the digestion process, 1% trifluoroacetic acid was added, and the mixture was then centrifuged.

#### Tandem mass tag labeling

Following the completion of the digestion and termination steps, the supernatant was subjected to desalting using a Sep-Pak C18 cartridge (Waters), and subsequently dried using a speedvac. Each individual sample was then reconstituted in 50 mM HEPES (pH 8.5) and labeled with either 11-plex or 16-plex TMT reagents. These labeled samples were mixed in equal proportions and subjected to another round of desalting in preparation for subsequent fractionation. A protein amount of 0.1 mg was used for each sample. The overall experimental design included a total of 2 batches of 11-plex TMT experiments and 3 batches of 16-plex TMT experiments.

#### Extensive two-dimensional LC/LC-MS/MS

The pooled samples, labeled with TMT, were subjected to fractionation using offline basic pH reversed-phase chromatography (HPLC), followed by acidic pH reverse phase LC-MS/MS analysis. The offline basic HPLC was conducted according to previously published methods.^88^^,^^89^ In the 16-plex TMT batch, we generated 40 concatenated fractions, while in the 11-plex TMT batch, 36 concatenated fractions were generated. The offline LC run was carried out using an XBridge C_18_ column (3.5 μm particle size, 4.6 mm × 25 cm, Waters) with a 3-h gradient. The mobile phase consisted of buffer A (10 mM ammonium formate, pH 8.0) and buffer B (95% acetonitrile, 10 mM ammonium formate, pH 8.0).^86^

For the subsequent acidic pH LC-MS/MS analysis, each fraction was sequentially run on a column (75 μm x 15–30 cm, 1.9 μm C18 resin from Dr. Maisch GmbH) heated to 65°C to reduce backpressure. The column was interfaced with an Orbitrap Fusion and Q Exactive HF MS system (Thermo Fisher). Peptides were eluted using a 1.5-2-h gradient with buffer A (0.2% formic acid, 5% DMSO) and buffer B (buffer A supplemented with 65% acetonitrile). The MS settings included MS1 scans with a resolution of 60,000, an AGC target of 1 × 10^6^, and a maximal ion time of 100 ms. Additionally, 20 data-dependent MS2 scans were performed in the *m/z* range of 410–1600, with a resolution of 60,000, an AGC target of 1 × 10^5^, a maximal ion time of ∼10^5^ ms, and higher-energy collisional dissociation with 38% normalized collision energy. The isolation window was set to 1.0 *m/z* with a 0.2 *m/z* offset, and dynamic exclusion was applied for approximately 15 s.

#### Identification of proteins by database search with JUMP software

To improve the sensitivity and specificity, we performed peptide identification with the JUMP search engine.^76^ JUMP searched MS/MS raw data against a composite target/decoy database^90^ to evaluate FDR. The target rat protein sequences (29,940 entries) were downloaded from the UniProt database. The decoy database, generated by reversing the target sequences, was concatenated to the target database. FDR was estimated by calculating the ratio of the number of decoy matches to the number of target matches. Major parameters included precursor and product ion mass tolerance (±15 ppm), full trypticity, static mass shift for the TMT tags (+229.16293/+304.20715) and carbamidomethyl modification of 57.02146 on cysteine, dynamic mass shift for Met oxidation (+15.99491), maximal missed cleavage (*n* = 2), and maximal modification sites (*n* = 3). Putative PSMs were filtered by mass accuracy and then grouped by precursor ion charge state and filtered by JUMP-based matching scores (Jscore and ΔJn) to reduce protein FDR below 1%. If one peptide could be generated from multiple homologous proteins, based on the rule of parsimony, the peptide was assigned to the canonical protein form in the manually curated Swiss-Prot database.

#### Proteomic analysis of SH-SY5Y cell knockdown experiments

Liquid Chromatography coupled with Data-Independent Acquisition (DIA) method was used for proteomic analysis for the knockdown experiments. Solid-Phase Enhanced Sample Preparation was utilized for sample processing.^91^ Data acquisition was conducted on an Orbitrap Exploris 480 Mass Spectrometer. The DIA data were analyzed using DIA-NN software,^78^ with searches conducted against a species-specific protein database. The search parameters included tryptic digestion and a precursor mass tolerance of 10 ppm. False discovery rates were controlled at 1% for both peptide and protein levels. Quantitative analysis was performed using the normalized abundance values generated by DIA-NN.

#### Proteogenomic analysis

Sequence variants between SHR and BN-Lx strains were provided by the rat reference genome project.^43^ All variants were re-annotated with the ANNOVAR program.^74^ A total of 8,723 missense variants were extracted for the proteogenomics analysis. We used JUMPg, a proteogenomics pipeline, to detect variant peptides.

#### RNA-seq data analysis

Transcriptomics data were obtained from a previous study,^40^ which included a total of 94 samples from 30 HXB/BXH RI strains, as well as their parental strains, SHR and BN-Lx. The dataset consisted of expression levels for 18,385 genes and included 2–3 replicates per strain. To facilitate subsequent analysis, the mean expression values for each strain were calculated and recorded.

#### Genotype data

The genotype of HXB/BXH (Rnor6.0) was download from the web of GeneNetwork in 07/2022,^92^ (https://genenetwork.org/). The genotypes were annotated with the Rnor6.0 genomic assembly.^43^ A total of 10,465 genotypes from 30 rat strains were used for the QTL mapping.

#### FAH knockdown experiment using siRNA transfection of SH-SY5Y cells

The Neon Transfection System (Invitrogen) was employed to transfect SH-SY5Y cells (ATCC) with 5 μM of either control siRNA or siRNA targeting FAH (sc-62356, Santa Cruz). Briefly, 0.5 million SH-SY5Y cells were washed with DPBS, resuspended in Buffer T containing the respective siRNA, and electroporated under the conditions of 1200 V, 20 ms, and 1 pulse. The electroporated cells were immediately transferred into 10 cm dishes and cultured in F12 medium (Cytiva) containing 10% FBS for 3 days, followed by whole proteome profiling.

### Quantification and statistical analysis

#### Protein quantification by JUMP software suite

Protein quantification was carried out using the following steps.^93^ We first extracted TMT reporter ion intensities of each PSM and corrected the raw intensities based on isotopic distribution of each labeling reagent. We discarded PSMs with low intensities (i.e., the minimum intensity of 1,000 and median intensity of 5,000). After normalizing abundance with the trimmed median intensity of all PSMs, we calculated the mean-centered intensities across samples (e.g., relative intensities between each sample and the mean) and summarized protein relative intensities by averaging related PSMs. Finally, we derived protein absolute intensities by multiplying the relative intensities by the grand mean of the three most highly abundant PSMs. Log_2_-transformed data were used for the subsequent normalized analysis.

#### Principal component analysis

PCA was used to visualize the differences among samples. All gene and metabolite abundance were used as features of PCA. The pairwise Euclidean distance between features was calculated. PCA was performed using the R package prcomp (version 3.4.0).^94^

#### Differential expression analysis

DEPs between the two strains were identified using the limma R package (version 3.46.0).^39^ The Benjamini-Hochberg method for FDR correction was used, and proteins with an adjusted *p* value <0.01 and fold change >2 were defined as differentially expressed between the SHR and BN-Lx parental strains.

#### Pathway enrichment

To assess the functional relevance of the DEPs, the R package clusterProfiler (version 3.18.1)^75^ was used for KEGG enrichment analysis. KEGG terms with a Benjamini-Hochberg *p* value <0.05 were defined as significantly enriched.

#### Analysis of the heritability of protein expression

Heritability of protein expression was determined by assessing the proportion of the total variance that can be attributed to additive genetic effects. The total variance was computed as the sum of squared differences from the mean expression for all 62 samples, whereas the additive variance was calculated by summing the square differences from the mean expression for all 31 rat strains after averaging data from two biological replicates (i.e., male and female for each strain).

#### Linkage analysis

For each protein, we estimated the proportion of variance in protein expression levels explained by all SNPs using a linear mixed model in GEMMA^47^ that corrects for kinship. GEMMA is the primary mapper in GeneNetwork.org.^36^^,^^37^^,^^38^ The top variant was selected as the QTL for the protein/gene eQTLs/pQTLs were defined as *cis* (local) if the QTL was within 1 Mb on either side of the TSS, whereas eQTLs/pQTLs were defined as *trans* (distal) if the peak association was at least 5 Mb outside of the exon boundaries.^47^^,^^95^

We permuted the sample labels ten times and applied the same pQTL mapping procedure to obtain an empirical null distribution of gene-level *p* values.^96^^,^^97^^,^^98^ After each permutation, we kept the most significant *p* value for each protein. With the empirical null distribution, we computed the FDR associated with each *p* value threshold and selected the *p* value threshold that provided a 5% FDR control.

#### Bayesian network analysis

We examined the causal connections among genotypes, protein expression levels, and phenotypes using the Bayesian network modeling tool (BNW, compbio.uthsc.edu/BNW).^55^^,^^56^ In this study, the BNW on GeneNetwork was employed with genotypes linked to a specific phenotype, proteins exhibiting pQTLs within the phenotypic QTL region, and phenotypic data as input variables. The analysis tests the causal dependencies between genetic variations, protein abundance, and phenotypic traits using Bayesian network.

#### Functional annotation of QTLs

ANNOVAR^74^ was used to functionally annotate the leading SNP of a QTL. RefSeq from the UCSC genome browser database was used to annotate SNPs. The functional consequence (synonymous, non-synonymous) of coding SNP was also determined.

#### Colocalization analysis

We utilized the coloc R package^77^ to assess the colocalization signals between *cis*-eQTLs and *cis*-pQTLs. A window of 500 kb was applied on both flanks of the pQTL locus. The coloc program was employed to derive posterior probabilities for five distinct yet mutually exclusive hypotheses. These hypotheses include: (i) no connection between any variant within the region and either *cis*-pQTL or *cis*-eQTL (H0); (ii) exclusive linkage with *cis*-pQTL excluding *cis*-eQTL (H1); (iii) exclusive linkage with *cis*-eQTL excluding *cis*-pQTL (H2); (iv) presence of two distinct QTLs (H3); and (v) presence of a shared QTL influencing both gene and protein expression (H4). A PP4 value exceeding 0.8 was considered a significant indication of colocalization.

#### Rat pangenome analysis

Gene pangenome graphs were built using the Pangenome Graph Builder (PGGB)^57^ that include wfmash alignments,^99^ and SEQWISH^58^ to produce unbiased graph models that losslessly represent both sequences and their variation. We then used ODGI,^59^ VG^100^ and Bandage^101^ to analyze the graph and produce pangenome visualizations that helped us zoom in gene candidates and structural variants.

## Supplemental information

Document S1. Figures S1–S6

Table S1. TMT-LC/LC-MS/MS-based proteomic profile of whole brain tissues from HXB/BXH rat strains, related to Figure 2

Table S2A. Variant peptides detected by JUMPg proteogenomics pipeline, related to Figure 2 Table S2B. Differentially expressed proteins (DEPs) identified between the two parental strains of the HXB/BXH RI rat panel, related to Figure 2 Table S2C. Enrichment analysis of proteins with higher expression in SHR compared to BN-Lx, related to Figure 2 Table S2D. Enrichment analysis of proteins with higher expression in BN-Lx compared to SHR, related to Figure 2 Table S2E. Enrichment analysis of proteins with variation coefficients, related to Figure 2

Table S3A. pQTLs identified from sex-averaged expression levels of 29 RI strains, related to Figure 3 Table S3B. Female-specific pQTLs identified in 29 RI strains, related to Figure 3 Table S3C. Male-specific pQTLs identified in 29 RI strains, related to Figure 3

Table S4. cis-eQTL identified in the rat brain tissue from 30 RI stains, related to Figure 4

Table S5A. Colocalization analysis for cis-pQTLs and cis-eQTLs, related to Figure 4 Table S5B. Colocalization analysis for cis-pQTLs and trans-pQTLs, related to Figure 5

Table S6. Phenotypic traits used for the linkage analysis, related to Figure 6

Table S7. Colocalization analysis for pQTL and traits, related to Figure 6
