# Supplementary material for: Genetic modulation of protein expression in rat brain
Source: iScience. 2025 Feb 21;28(3):112079. doi: 10.1016/j.isci.2025.112079 (PMC11930185; doi:10.1016/j.isci.2025.112079)
Supplement: Document S1. Figures S1–S6 [file mmc1.pdf]

iScience, Volume *28*

Supplemental information

Genetic modulation of protein expression in rat brain

Ling Li, Zhiping Wu, Andrea Guarracino, Flavia Villani, Dehui Kong, Ariana Mancieri, Aijun Zhang, Laura Saba, Hao Chen, Hana Brozka, Karel Vales, Anna N. Senko, Gerd Kempermann, Ales Stuchlik, Michal Pravenec, Joseph Lechner, Pjotr Prins, Ramkumar Mathur, Lu Lu, Kai Yang, Junmin Peng, Robert W. Williams, and Xusheng Wang

Supplemental Information

Genetic Modulation of Protein Expression in Rat Brain

Ling Li, Zhiping Wu, Andrea Guarracino, Flavia Villani, Dehui Kong, Ariana Mancieri, Aijun Zhang, Laura Saba, Hao Chen, Hana Brozka, Karel Vales, Anna N. Senko, Gerd Kempermann, Ales Stuchlik, Michal Pravenec, Joseph Lechner, Pjotr Prins, Ramkumar Mathur, Lu Lu, Kai Yang, Junmin Peng, Robert W. Williams,and Xusheng Wang

Supplemental Figures

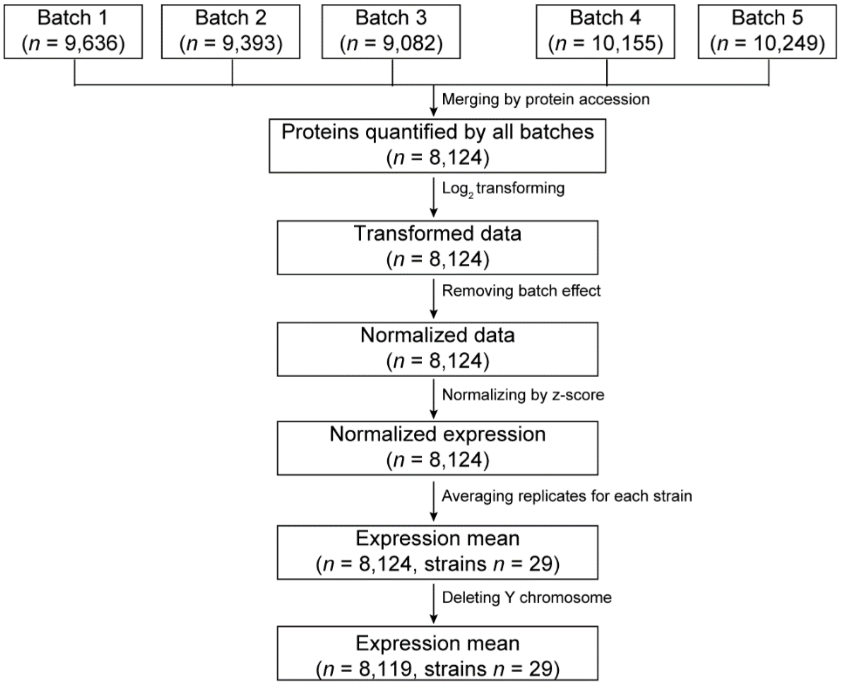

Figure S1. Workflow for the analysis of quantitative proteomics data, related to Figure 2.

A total of five batches of TMT experiments were conducted, generating quantitative proteomics data for 62 samples. Proteins from each batch were merged based on their Swiss-Prot/TrEMBL protein accession numbers. The protein intensity values were $\log_2$ transformed, followed by normalization across the five batches using the batch removal function in the LIMMA package. The normalized expression data were further transformed using Z-score procedure. The expression data were averaged across both sexes for each strain. Any proteins from Y chromosome were excluded from the dataset.

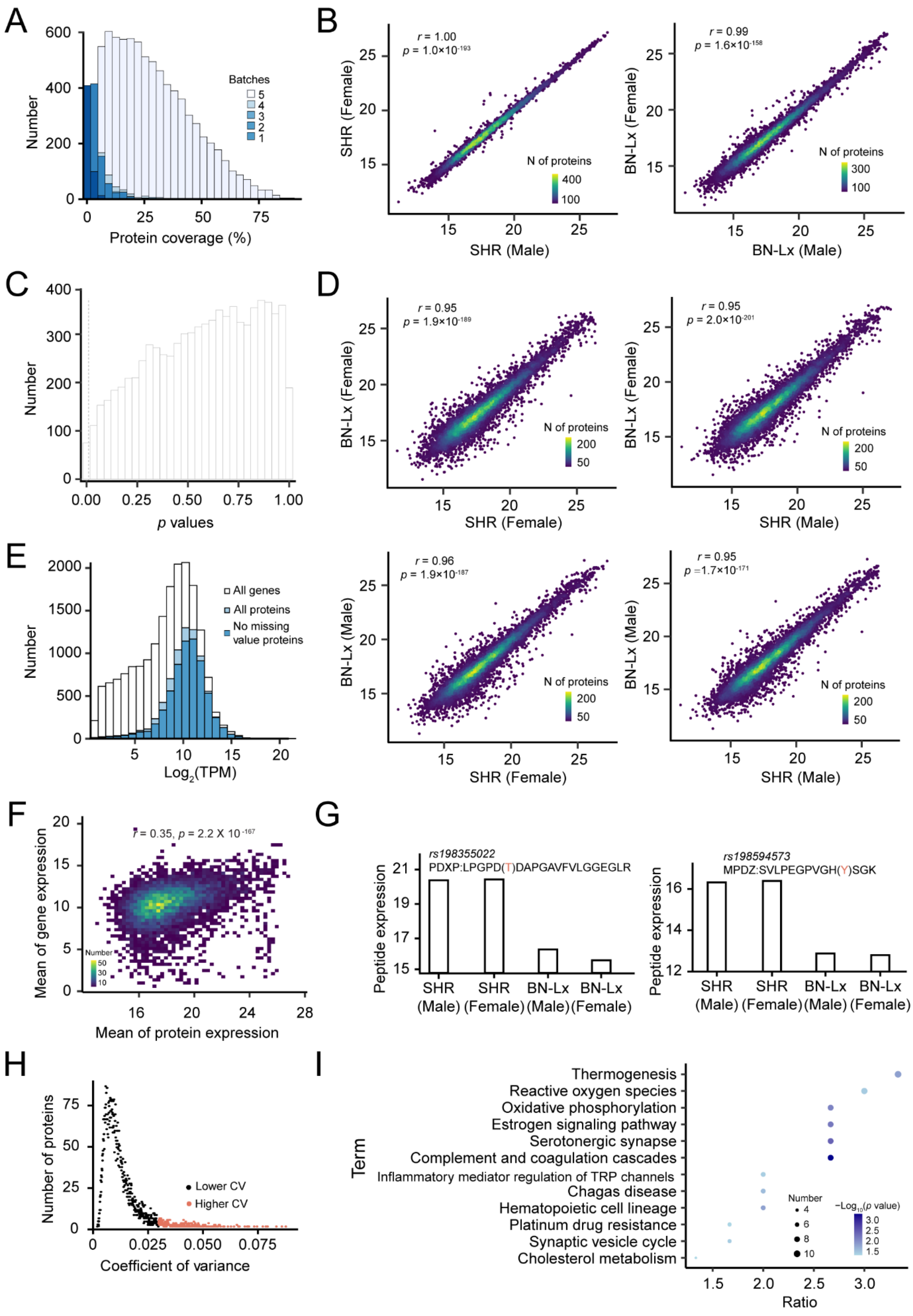

A
Number
Protein coverage (%)
Batches
5
4
3
2
1
B
r = 1.00
p = 1.0×10-193
SHR (Female)
SHR (Male)
N of proteins
r = 0.99
p = 1.6×10-158
BN-Lx (Female)
BN-Lx (Male)
C
Number
p values
D
r = 0.95
p = 1.9×10-189
BN-Lx (Female)
SHR (Female)
r = 0.95
p = 2.0×10-201
SHR (Male)
E
Number
Log2(TPM)
All genes
All proteins
No missing value proteins
r = 0.96
p = 1.9×10-187
BN-Lx (Male)
r = 0.95
p =1.7×10-171
F
r = 0.35, p = 2.2 X 10-167
Mean of gene expression
Mean of protein expression
Number
G
rs198355022
PDXP:LPGPD(T)DAPGAVFVLGGEGLR
Peptide expression
SHR (Male)
SHR (Female)
BN-Lx (Male)
BN-Lx (Female)
rs198594573
MPDZ:SVLPEGPVGH(Y)SGK
H
Number of proteins
Lower CV
Higher CV
Coefficient of variance
I
Thermogenesis
Reactive oxygen species
Oxidative phosphorylation
Estrogen signaling pathway
Serotonergic synapse
Complement and coagulation cascades
Inflammatory mediator regulation of TRP channels
Chagas disease
Hematopoietic cell lineage
Platinum drug resistance
Synaptic vesicle cycle
Cholesterol metabolism
Term
Number
−Log10(p value)
Ratio

Figure S2. Rat brain proteome analysis: coverage, correlation, and variability across parental strains, related to Figure 2.

(A) Histogram showing the coverage of quantified proteins across 29 batches of TMT experiments. The open bar represents the distribution of proteins quantified by all five TMT batches, while the blue gradient color shows the distribution of proteins detected in 1 to 4 TMT batches. Protein coverage is defined as the percentage of amino acids identified by the proteomics data. (B) Scatter plot displaying correlations between male and female of the SHR and BN-Lx strains. (C) Distribution of *p* values from the statistical tests comparing male and female samples for all proteins. (D) Scatter plots showing the correlations between the SHR and BN-Lx strains. (E) Histogram showing the coverage of proteomic data compared to RNA-seq data. The open bar represents the distribution of protein-coding genes detected by RNA-seq, the light blue bar indicates the distribution of protein-coding genes from proteomic data, and the navy bar indicates the distribution of protein-coding genes from no missing value proteomic data. (F) Scatter plot showing a comparison of gene expression levels and protein abundance. The expression is defined as the average expression across all samples. (G) Expression levels of variant peptides in SHR and BN-Lx strains. The amino acid highlighted in red within the parenthesis represents the reference amino acid encoded by the allele from the reference BN-Lx genome. (H) Distribution of coefficient of variation (CV) for all proteins across 29 strains. (I) Enriched KEGG pathways in the highly variable proteins.

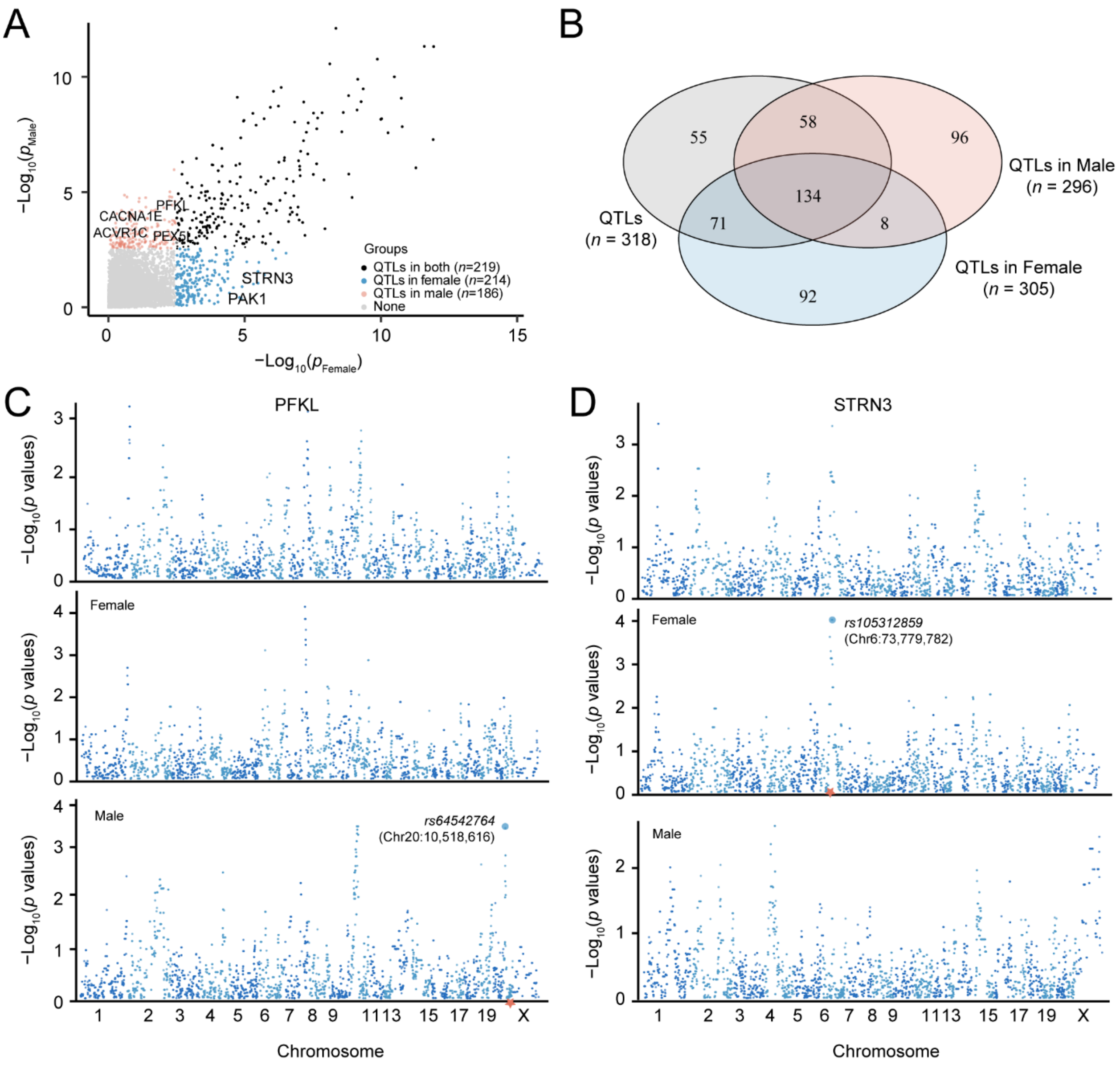

Figure S3. Sex-specific genetic regulation of the brain proteome, Related to Figure 3.

(A) Scatter plot illustrating the significance of QTLs identified in both sexes, with QTLs specific to females (blue), males (red), and those found in both (black). (B) Venn diagram showing the overlap and distinct QTLs identified in male and female linkage analyses, as well as those common to both. (C) Manhattan plots display the genome-wide *p*-values for associations between SNPs and PFKL protein expression, separated by sex. The top *cis*-pQTL (*rs64542764*) for PFKL protein expression in males is indicated by a red diamond. (D) Manhattan plots display the genome-wide *p*-values for associations between SNPs and STRN3 protein expression, separated by sex. The top *cis*-pQTL (*rs105312859*) for STRN3 protein expression in females is indicated by a red diamond.

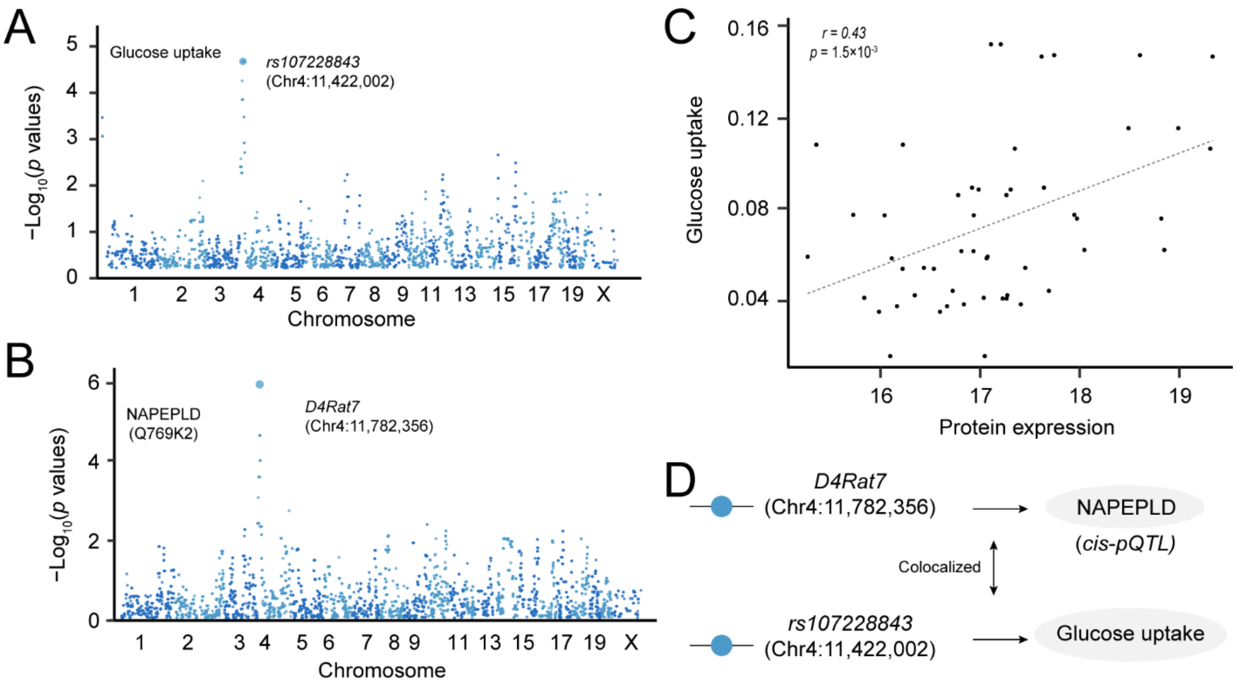

Figure S4. Co-localization of *cis*-QTLs and phenotypic traits, Related to Figure 6.

(A-B) Manhattan plot showing a colocalized QTL associated with a phenotypic trait, glucose uptake (A), and associated with NAPEPLD protein expression (B). (C) Scatter plot correlating NAPEPLD expression with glucose uptake level. (D) Schematic representation illustrating the pleiotropic effect of the genotype on both protein expression and glucose uptake levels.

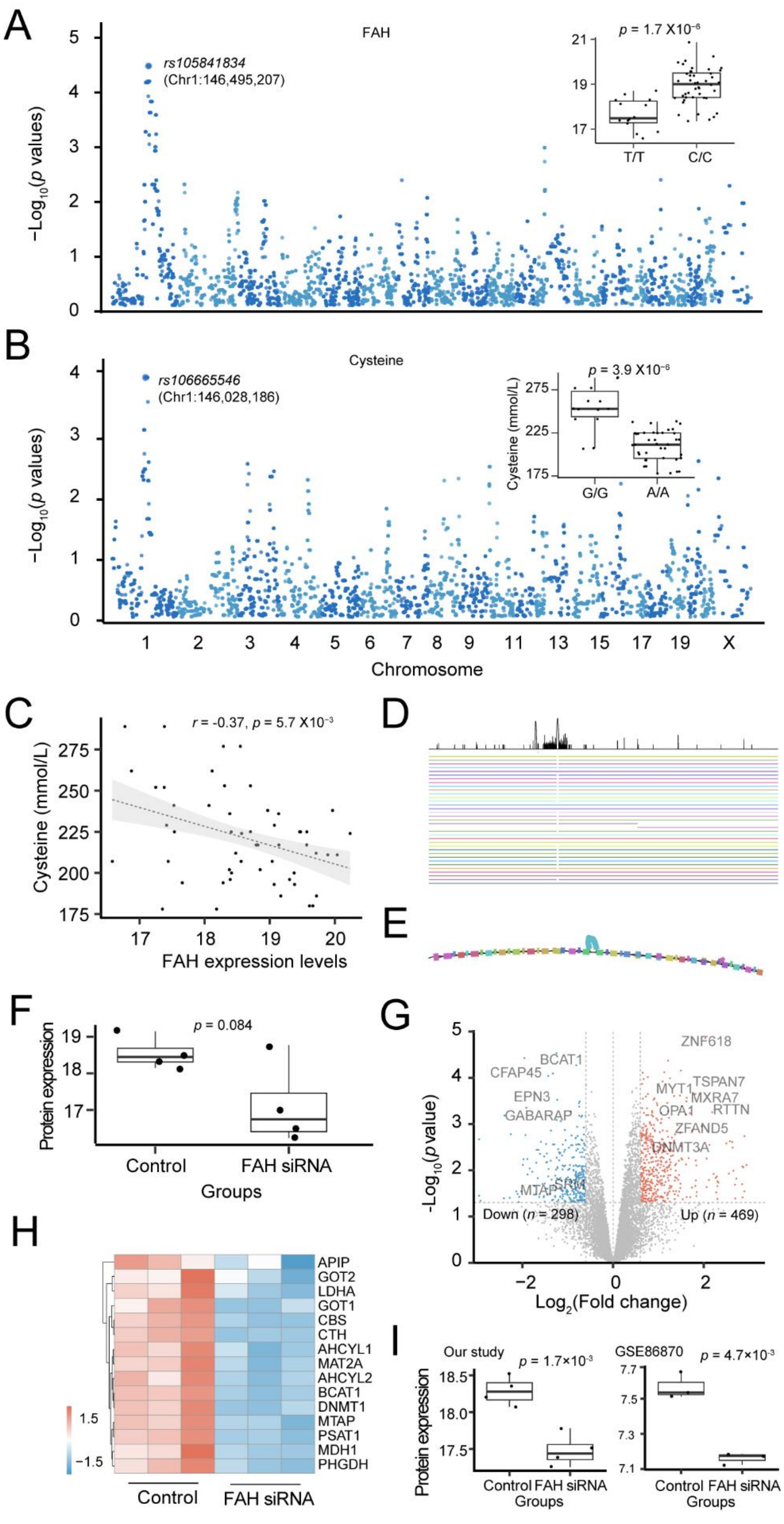

A
FAH
rs105841834
(Chr1:146,495,207)
p = 1.7 X10⁻⁶
T/T
C/C
−Log10(p values)
B
Cysteine
rs106665546
(Chr1:146,028,186)
p = 3.9 X10⁻⁶
Cysteine (mmol/L)
G/G
A/A
Chromosome
C
r = -0.37, p = 5.7 X10⁻³
Cysteine (mmol/L)
FAH expression levels
D
E
F
p = 0.084
Protein expression
Control
FAH siRNA
Groups
G
ZNF618
BCAT1
CFAP45
TSPAN7
MYT1
MXRA7
EPN3
OPA1
RTTN
GABARAP
ZFAND5
DNMT3A
MTAP
Down (n = 298)
Up (n = 469)
-Log10(p value)
Log2(Fold change)
H
APIP
GOT2
LDHA
GOT1
CBS
CTH
AHCYL1
MAT2A
AHCYL2
BCAT1
DNMT1
MTAP
PSAT1
MDH1
PHGDH
1.5
−1.5
Control
FAH siRNA
I
Our study
p = 1.7×10⁻³
GSE86870
p = 4.7×10⁻³
Protein expression
Control
FAH siRNA
Groups

Figure S5. Genetic variants associated with FAH protein expression and validation by knockdown experiments, Related to Results.

(A) Manhattan plot depicting genome-wide association of the locus *rs105841834* associated with Fah protein expression, box plots showing the distribution of trait values by genotype. (B) Manhattan plot depicting genome-wide association of the locus *rs106665546* associated with blood cysteine levels, box plots showing the distribution of trait values by genotype. (C) Scatter plot correlating FAH protein expression with blood cysteine levels. (D) Genome browser view displaying genetic variants within the Fah gene, with an evident large insertion highlighted in SHR and some HXB strains. (E) Schematic arc plot visualizing the structural variation of Fah genes across SHR and HXB/BXH strains. (F) Boxplot showing FAH expression levels in FAH knock down samples compared to controls. (G) Volcano plot displaying the differentially expressed proteins. (H) Heatmap showing the genes involved in cysteine and methionine metabolism pathway in the previous study. (I) Boxplot showing BCAT1 expression levels in FAH knockdown samples compared to controls.

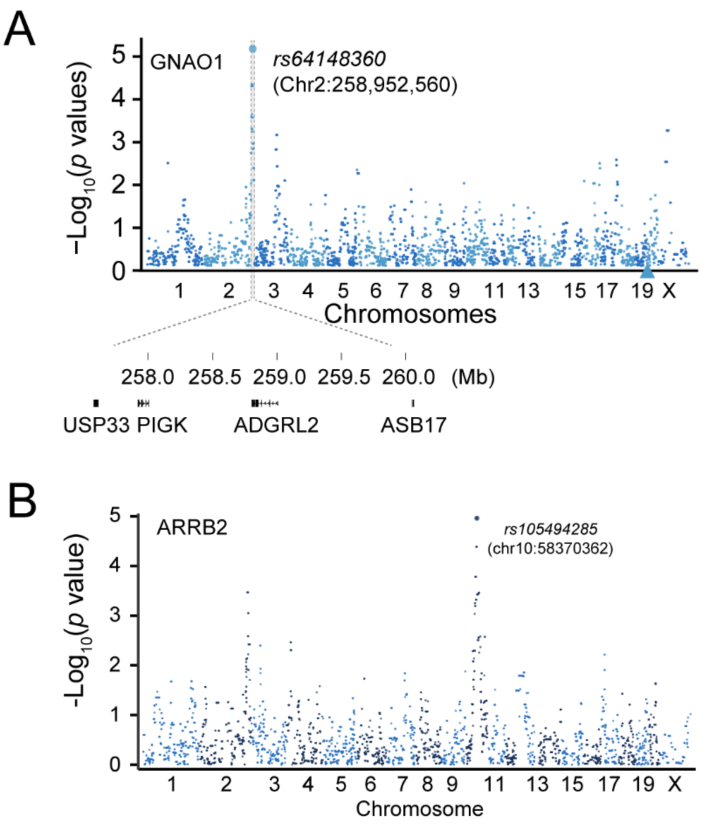

Figure S6. Manhattan plots illustrating genetic regulation of protein expression in rats, highlighting associations relevant to human diseases, Related to Results.

(A) Manhattan plot showing a *trans*-pQTL (i.e., rs64148360) associated with GNAO1 protein expression. (B) Manhattan plot showing a *cis*-pQTL (i.e., rs105494285) associated with ARRB2 protein expression.

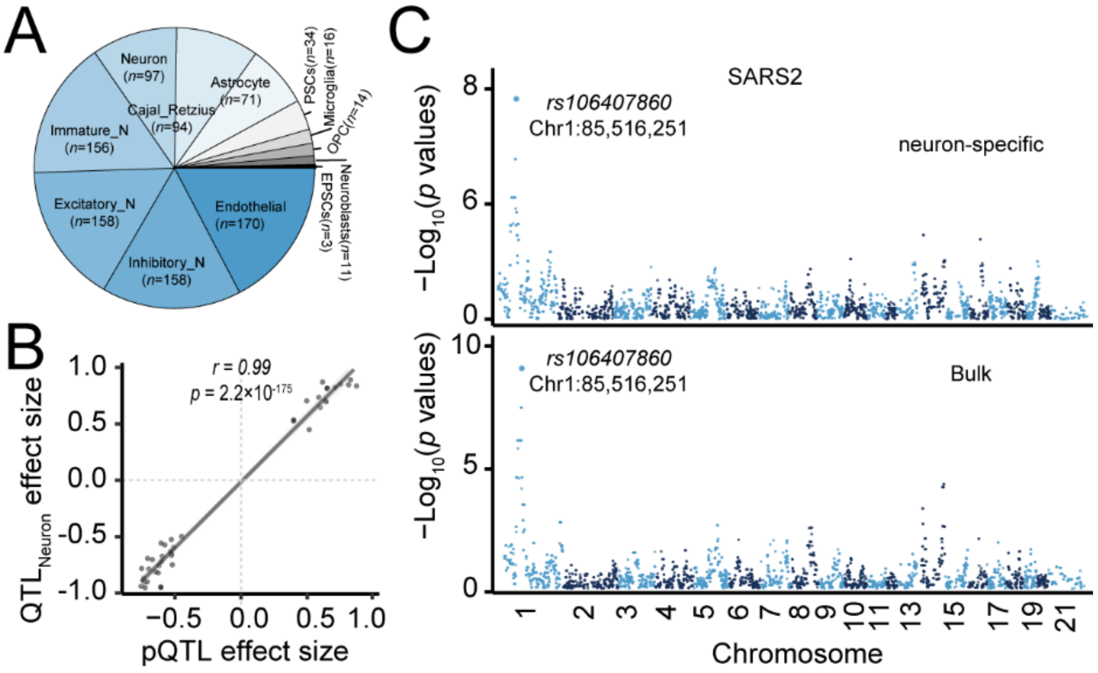

Figure S7. Analysis of cell-type-specific QTLs, Related to Discussion.

(A) Number of QTLs specific to each cell type. (B) Correlation of effect sizes between neuron-specific and bulk QTLs. (C) Manhattan plots depicting a QTL that colocalizes in both bulk and neuron datasets, regulating SARS2 protein expression.
